# Automated literature surveillance in anesthesiology: an open, customizable dashboard

**DOI:** 10.1016/j.bjane.2026.844833

**Published:** 2026-09-19

**Authors:** Heleno de Paiva Oliveira

**Affiliations:** Universidade Federal do Rio Grande do Norte (UFRN), Departamento de Cirurgia, Natal, RN, Brazil

Dear Editor,

Keeping up with medical literature is challenging for clinicians and educators. Publication growth and dispersion have encouraged alerts, curated resources, and digital tools for current awareness.^1^^,^^2^ In this context, the Anesthesia Table of Contents (TOC) Dashboard was developed to bring recent anesthesiology and pain medicine publications into one searchable, browser-based interface. Users can adapt its source collection to their interests.

The project builds on an established ecosystem: TOC aggregation exemplified by JournalTOCs; institutional journal browsing through BrowZine; saved-search alerts from PubMed/MyNCBI and Google Scholar; personalized medical-literature feeds from Read by QxMD; and citation-based discovery through ResearchRabbit. Machine-learning approaches such as LitSuggest have also been described.^3^ The dashboard does not claim a new aggregation concept or superiority over these alternatives. Its incremental contribution combines specialty-focused defaults, account-free browsing, local bookmarking, and openly modifiable source-selection and update workflows.

For this revision, a 31-source selection is specified using the 2025 SCImago Journal Rank (SJR)^4^ export: 30 journals and the book series Advances in Anesthesia. Entries were retained when classified exclusively under “Anesthesiology and Pain Medicine” in the first or second quartile (14 Q1 and 17 Q2 sources). This reproducible starting selection excludes titles assigned to multiple subject categories and is not an exhaustive PubMed journal list. Sources are identified by the International Standard Serial Number (ISSN).

The Q1 sources are British Journal of Anaesthesia; Anaesthesia; European Journal of Anaesthesiology; Anesthesiology; Journal of Clinical Anesthesia; Korean Journal of Anesthesiology; Pain Reports; Anesthesia & Analgesia; BJA Open; Pain Physician; Canadian Journal of Pain; Korean Journal of Pain; Pain Practice; Journal of Anesthesia.

The Q2 sources are BJA Education; BMC Anesthesiology; Journal of Pain Research; Best Practice and Research in Clinical Anaesthesiology; Minerva Anestesiologica; Current Opinion in Anesthesiology; British Journal of Pain; Local and Regional Anesthesia; Brazilian Journal of Anesthesiology; Anesthesia and Pain Medicine (ISSN 1975-5171); Indian Journal of Anaesthesia; Pain Management; Interventional Pain Medicine; Anesthesiology and Pain Medicine (ISSN 2228-7523); Current Anesthesiology Reports; Advances in Anesthesia; Saudi Journal of Anaesthesia.

Customization is central to the design. Users can create an independent repository copy, known as a fork, and edit *sources.json* to add or remove journals by name and ISSN. Deployments can include other PubMed-indexed journals, titles assigned to multiple categories, lower-quartile journals, or locally relevant publications when suitable Crossref metadata are available. This changes the retrieved collection, whereas the interface’s journal filter only narrows the existing dataset. Browsing requires no setup; customization requires repository configuration and enabling update and hosting workflows. Specifying a source does not establish complete retrieval of its publications.

A scheduled GitHub Actions workflow retrieves Crossref metadata by ISSN, standardizes records, and removes duplicates using Digital Object Identifiers (DOIs), with article links as a fallback. Records include titles, authors, dates derived from publication and metadata timestamps, and publisher links when available. Ahead-of-print status is inferred rather than independently verified. Matching PubMed links provide access to abstracts and related resources.^5^ Links are added through the National Center for Biotechnology Information E-utilities, subject to lookup limits. The build produces a static dataset; the browser does not harvest upstream metadata (Fig. 1).

**Figure 1:**
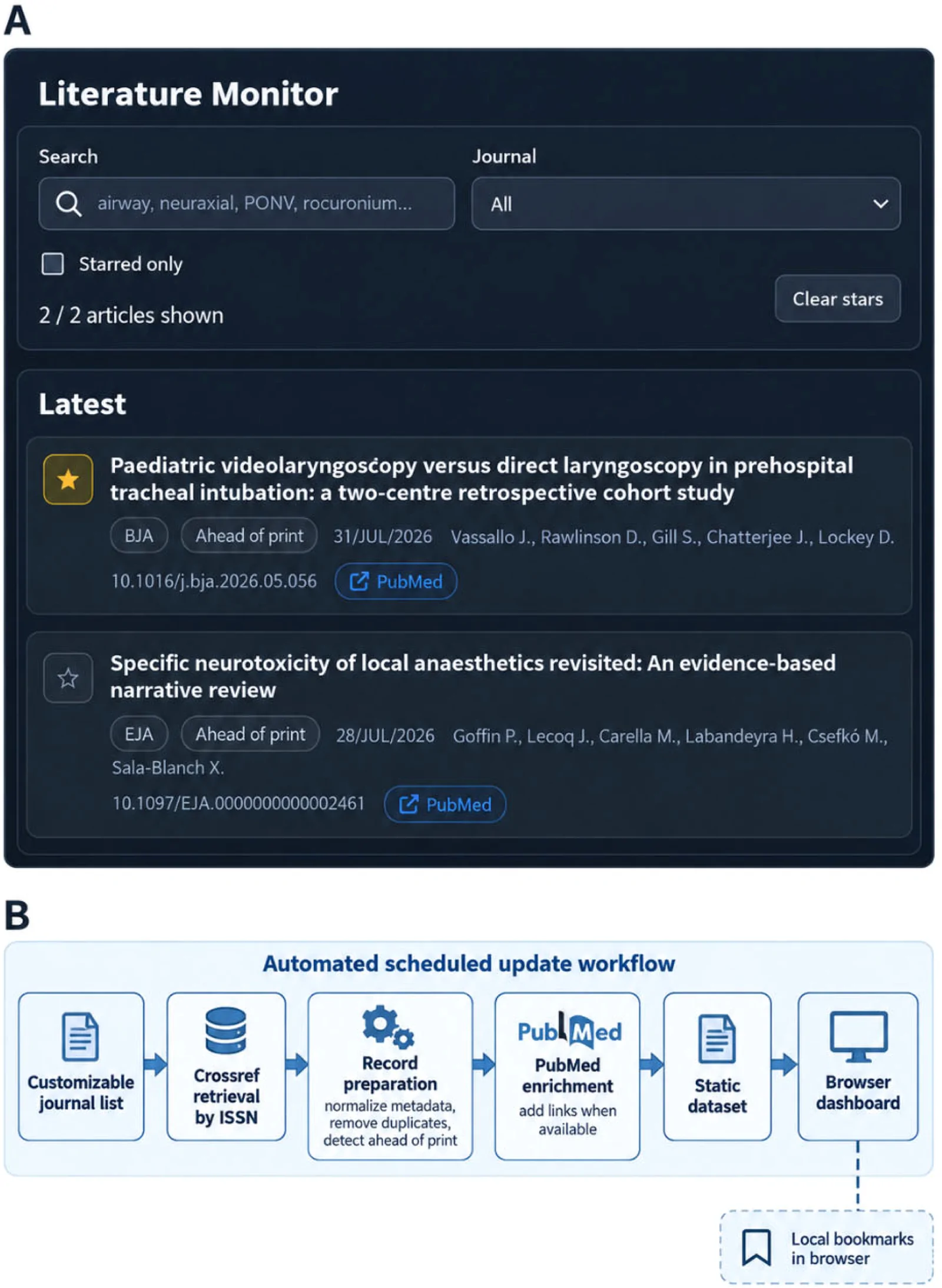
Metadata.

The interface provides keyword search across titles, authors, journal names, and DOIs, journal filtering, progressive loading, and bookmarking. Search and filtering run locally; bookmarks remain in the user’s browser. Browsing requires neither a dashboard account nor institutional authentication. The supplied application contains no analytics scripts, although external services may retain their own access logs. Static hosting avoids a dedicated runtime application backend, not dependence on hosting and build services. Source files and workflow configuration are inspectable. Update schedules and retrieval limits are configurable; interface-language changes require editing the source code.

A residency program could adapt the source list to its curriculum and select articles for journal clubs; clinicians could maintain subspecialty-focused deployments. Account-free browsing may lower access barriers where institutional aggregation services are unavailable, including teaching settings in Brazil and elsewhere. These are potential applications, not demonstrated benefits. The dashboard does not bypass publisher restrictions on full-text access.

No formal usability testing, adoption assessment, comparative evaluation, or measurement of educational or clinical impact has been performed. Coverage and timeliness depend on upstream metadata, retrieval limits, and successful builds. Administrative metadata updates may affect chronology; dates, ahead-of-print labels, and PubMed links may be incomplete or inaccurate. An absent PubMed link does not establish non-indexing. Bookmarks are browser-specific and unsynchronized. Maintenance is required, and reliable offline reloading is not provided. These limitations preclude claims of comprehensive surveillance, demonstrated time savings, or improved learning.

The dashboard is available at https://helenopaiva.github.io/AnesthesiaTOC/, with source code at https://github.com/HelenoPaiva/AnesthesiaTOC. The MIT License permits modification and redistribution. This adaptable starting point for literature awareness does not replace bibliographic databases, systematic searches, or critical appraisal. Future work should assess usability, customization effort, and sustained use in journal-club workflows before evaluating educational benefit. Sharing the interface and workflow invites anesthesiologists and educators to shape the collection and its evaluation.

## Data availability statement

Source code is publicaly available at https://github.com/HelenoPaiva/AnesthesiaTOC.

## Ethics approval and informed consent

Not applicable. This report describes software using open bibliographic metadata and involves no patient data.

## Clinical trial registration

Not applicable.

## Declaration of generative AI and AI-assisted technologies in the writing process

During manuscript preparation, the author used ChatGPT (OpenAI) to assist with drafting, language revision, and reference identification. ChatGPT also assisted with debugging portions of the software and preparing code for the revised figure. Figure 1 uses a browser-rendered interface with fixed bibliographic input and a programmatically drawn workflow; the earlier image-generated illustration is not retained. The author reviewed and edited the AI-assisted material, checked the references and technical statements, and takes full responsibility for the content of the submitted manuscript.

## Author’ contributions

Heleno de Paiva Oliveira: Conceptualization; methodology; software; writing-original draft; writing-review & editing.

## Funding

This research did not receive any specific grant from funding agencies in the public, commercial, or not-for-profit sectors.

## Conflicts of interest

The author is the developer and owner of Anesthesia TOC Dashboard, registered as a computer program at the Brazilian National Institute of Industrial Property (INPI; BR512026000086-6). The software is open source and free to use; the author derives no financial benefit from its distribution.
